# Cdx1 and Gsc distinctly regulate the transcription of BMP4 target gene *ventx3.2* by directly binding to the proximal promoter region in *Xenopus* gastrulae

**DOI:** 10.1016/j.mocell.2024.100058

**Published:** 2024-03-23

**Authors:** Ravi Shankar Goutam, Vijay Kumar, Unjoo Lee, Jaebong Kim

**Affiliations:** 1 Department of Biochemistry, Institute of Cell Differentiation and Aging, College of Medicine, Hallym University, Chuncheon, Gangwon-Do 24252, Korea; 2 Laboratory of Regenerative Medicine, College of Pharmacy, Ewha Womans University, Seoul, Korea; 3 Department of Electrical Engineering, Hallym University, Chuncheon, Gangwon-Do 24252, Korea

**Keywords:** Caudal type homeobox 1, Goosecoid, ventx3.2 promoter, Transcriptional regulation, Xenopus

## Abstract

A comprehensive regulatory network of transcription factors controls the dorsoventral patterning of the body axis in developing vertebrate embryos. Bone morphogenetic protein signaling is essential for activating the Ventx family of homeodomain transcription factors, which regulates embryonic patterning and germ layer identity during *Xenopus* gastrulation. Although Ventx1.1 and Ventx2.1 of the *Xenopus* Ventx family have been extensively investigated, Ventx3.2 remains largely understudied. Therefore, this study aimed to investigate the transcriptional regulation of *ventx3.2* during the embryonic development of *Xenopus*. We used goosecoid (Gsc) genome-wide chromatin immunoprecipitation-sequencing data to isolate and replicate the promoter region of *ventx3.*2. Serial deletion and site-directed mutagenesis were used to identify the *cis*-acting elements for Gsc and caudal type homeobox 1 (Cdx1) within the *ventx3.2* promoter. Cdx1 and Gsc differentially regulated *ventx3.2* transcription in this study. Additionally, positive *cis*-acting and negative response elements were observed for Cdx1 and Gsc, respectively, within the 5′ flanking region of the *ventx3.2* promoter*.* This result was corroborated by mapping the active Cdx1 response element (CRE) and Gsc response element (GRE). Moreover, a point mutation within the CRE and GRE completely abolished the activator and repressive activities of Cdx1 and Gsc, respectively. Furthermore, the chromatin immunoprecipitation-polymerase chain reaction confirmed the direct binding of Cdx1 and Gsc to the CRE and GRE, respectively. Inhibition of Cdx1 and Gsc activities at their respective functional regions, namely, the ventral marginal zone and dorsal marginal zone, reversed their effects on *ventx3.2* transcription. These results indicate that Cdx1 and Gsc modulate *ventx3.2* transcription in the ventral marginal zone and dorsal marginal zone by directly binding to the promoter region during *Xenopus* gastrulation.

## Highlights

• Cdx1 and Gsc differentially regulate *ventx3.2* transcription.
• Gsc limits *ventx3.2* transcription in the dorsal marginal zone.
• Cdx1 positively regulates *ventx3.2* transcription in the ventral marginal zone.
• Cdx1 and Gsc directly bind to the *ventx3.2* promoter during *Xenopus* gastrulation.

## GRAPHICAL ABSTRACT

**Figure:**
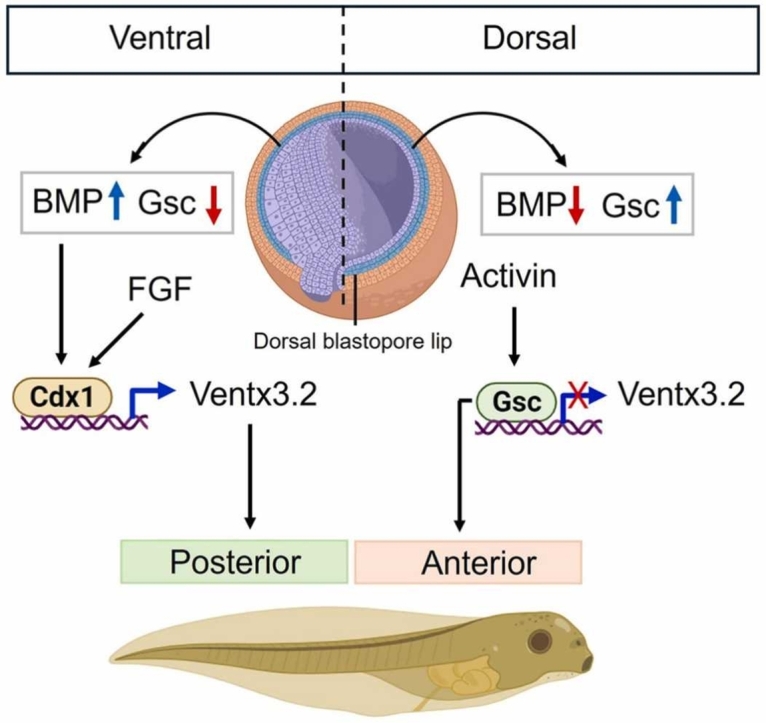

The regulation of embryonic development is governed by critical signaling that occurs in a certain spatial and temporal pattern. Bone morphogenetic protein 4 (Bmp4) expression is predominantly localized in the ventral region, where it regulates the development of ventral and posterior tissues via the activation of the *ventx* gene family. The fibroblast growth factor downstream Cdx1 bound to *ventx3.2* promoter and stimulate the expression in the ventral mesodermal region. Conversely, the activin signaling remains high in the dorsal mesodermal zone, regulating the expression of Bmp antagonist gene such goosecoid (*gsc*). Where, Gsc strongly suppresses the activity of *bmp4* and *ventx3.2* gene to sustain the development of anterior differentiation.

## INTRODUCTION

Bone morphogenetic protein 4 (BMP4), which belongs to the transforming growth factor β family, plays numerous roles in embryonic development. It regulates the initial development of vertebrates by acting as an early morphogen that determines ectoderm and ventral mesoderm differentiation and dorsoventral (DV) patterning (Dosch et al., 1997, Tucker et al., 2008, Wills et al., 2008). This protein directly activates the expression of *ventx* family members, namely, *ventx1.1*, *ventx1.2*, *ventx2.1*, *ventx2.2*, *ventx3.1*, and *ventx3.2* (Henningfeld et al., 2002, Lee et al., 2011, Shapira et al., 1999, Kumar et al., 2022). Given its association with changes in the developmental fate of embryos, Ventx1.1 is considered a major downstream target of BMP4. A ventralized *Xenopus* embryo is produced via the ectopic expression of *Ventx1.1* (Hwang et al., 2002b). In contrast, the antimorphic ventx1.1 construct promotes neuralization and expands the expression domain of Spemann organizer-specific genes, such as Gsc and *Chordin* (Hwang et al., 2002b). Moreover, ventx2.1 regulates neural crest specification and ectomesenchyme formation in the ectoderm (Scerbo and Monsoro-Burq, 2020). In *Xenopus* embryos, morpholino-based *ventx2.1* knockdown results in the development of a partially duplicated secondary axis and enlargement of the Spemann organizer (Onichtchouk et al., 1996, Onichtchouk et al., 1998). Our previous investigation on the evolution of the ventx family in *Xenopus* (Kumar et al., 2022) revealed that Ventx1.1 and Ventx2.1 have a shared ancestor. In contrast, Ventx3.1 and Ventx3.2 are the most unique members of the Ventx cluster (Kumar et al., 2022). While the function of ventx3.1 remains unknown, limited information is available regarding that of ventx3.2. The BMP4/Smad1 signaling pathway targets *ventx3.2*, a downstream gene that is specifically expressed in the ventral marginal zones (VMZ) and ectodermal area during the gastrulation of *Xenopus* embryos (Shapira et al., 2000, Shapira et al., 1999). Additionally, the ectopic expression of Ventx3.2 in *Xenopus* restores the dominant negative Bmp4 receptor-based attenuation of BMP4 signaling. This leads to the dose-dependent ventralization of embryos (Shapira et al., 2000) and recapitulation of ventx1.1 function during gastrulation. This suggests that Ventx3.2 can restore BMP4 function in developing *Xenopus* embryos, making it a crucial ventx family member. Research utilizing antimorphs has shown that the Ventx3.2 antimorph can induce secondary axis formation, whereas partial loss of Ventx3.2 enhances the efficacy of secondary axis formation by the dominant negative Bmp receptor or Smad6 in *Xenopus laevis* (Shapira et al., 2000). These loss-of-function investigations show that Ventx3.2 plays a major role in limiting the Spemann organizer.

Genes regulating organizers can counteract BMP4 function (Sander et al., 2007, Steinbeisser et al., 1995). This interaction helps maintain a reciprocal regulatory network between genes specific to the dorsal and ventral regions (Fainsod et al., 1994, Graff et al., 1994, Sasai et al., 1995, Schmidt et al., 1995, Steinbeisser et al., 1995). Thus, it determines DV and anteroposterior patterning in *Xenopus* embryos (De Robertis and Sasai, 1996, Ferguson, 1996). Gsc negatively regulates the transcription of *BMP4* and its target genes (Sander et al., 2007, Steinbeisser et al., 1995) while activating that of organizer-specific genes in the dorsal region, such as *chrd* (Kumar et al., 2023). A Gsc-mediated genome-wide chromatin immunoprecipitation (ChIP)-sequencing analysis (data not shown) revealed that Gsc selectively binds to the promoter region of *ventx3.2*. However, the molecular mechanism through which Gsc regulates *ventx3.2* transcription remains unknown.

Fibroblast growth factor (FGF) signaling interacts with BMP/smad1 and can activate and target certain members of the ventx family (Kumar et al., 2019, Kumar et al., 2018). For example, Smad1 physically interacts with *Xenopus* brachyury (Xbra) to activate *ventx1.1* and *ventx2.1*. This interaction, which can occur when Smad1 and Xbra act together or separately (Kumar et al., 2018, Messenger et al., 2005), increases the degree of transcription of *ventx1.1* and *ventx2.1* in *Xenopus*. Cdx1, another member of the FGF signaling cascade, directly activates ventx family members during *Xenopus* gastrulation (Kumar et al., 2021a, Pillemer et al., 1998). *Xenopus* Cdx1 acts as a transcriptional activator to stimulate the expression of BMP4 and its target genes, including *ventx1.1*, *ventx1.2*, *ventx2.1*, and *Xpo*. Moreover, it suppresses the expression of organizer-specific genes, such as *Gsc* and *Otx* (Pillemer et al., 1998). Direct binding of Cdx1 to the 5′ promoter region of *ventx1.1* activates transcription (Kumar et al., 2019). However, whether Cdx1 activates *ventx3.2* transcription remains unclear.

In this study, we aimed to investigate the transcriptional regulation of *ventx3.2* during the embryonic development of *Xenopus*. We hypothesized that Gsc suppresses *ventx3.2* expression in the gastrula to preserve dorsal identity, whereas Cdx1 enhances *ventx3.2* transcription to retain the identity of the ventral mesoderm. Our findings provide novel insights into the complex molecular mechanisms underlying the regulation of *ventx3.2* transcription in the ventral and dorsal mesoderm during *Xenopus* gastrulation.

## MATERIALS AND METHODS

### Ethics Statement

All animal experiments adhered to the regulations established by the Hallym University Institutional Animal Care and Use Committee, as documented in Hallym 2012-76, 2013-130, and 2019-79. Each member of our research team participated in educational and training workshops on the appropriate handling and utilization of experimental animals. Adult *X. laevis* were maintained by personnel approved for laboratory animal care in accordance with the guidelines of the Institute of Laboratory Animal Resources of Hallym University. They were housed in suitable containers with a 12-hour light and dark cycle (LD 12:12 h) at 18 °C.

### Embryo Manipulation

The *X. laevis* used in this study were supplied by the Korean *Xenopus* Resource Center for Research. Embryos were generated using in vitro fertilization (IVF) after stimulating female frogs with 500 units of human chorionic gonadotropin purchased from LG (IVF-C 1000 international unit (IU)). DNA and messenger RNA (mRNA) constructs were injected into the animal poles of embryos at the 1-cell stage. Animal cap (AC) explants were extracted from embryos with or without injection at stage 8. These explants were then grown in L-15 medium until they reached the required stages for subsequent AC assays.

### DNA and RNA Preparation

pCS4-Myc-Cdx1 and pCS4-3Flag-Gsc mRNAs were created by linearizing the target vectors with the Not1 and *Acc*65I restriction enzymes, respectively. The linearized vectors (pCS4-Myc-Cdx1 and pCS4-3Flag-Gsc) were used during in vitro transcription tests, which were performed using the mMessage mMachine Kit (Ambion) according to the manufacturer’s instructions. The synthesized mRNAs were quantified using Thermo-scientific Nanodrop One and then diluted in diethyl pyrocarbonate-treated water to achieve a final concentration of 1 ng/5 nL. The diluted mRNA was stored at −80 °C for injections.

### Cloning of ventx3.2 Genomic DNA

The *Ventx3.2* promoter region at -1 to -1188 bp (JBrowse laevis 10.1: Chr7L:21729949-21731136) was cloned using pure genomic DNA obtained from *X. laevis*. The cloning process involved the utilization of specific primers (V.x3.2(-1188)-F and V.x3.2-R/ Table 1) designed for the amplification of DNA fragments. The amplicon of the ventx3.2 (-1188 bp) promoter was inserted into the pGL3-Basic plasmid (Promega) using the *Nhe*I and *Xho*I restriction sites as described previously (Kumar et al., 2023). The construct is henceforth referred to as “*ventx3.2(-1188)-luc*.”

**Table 1:** Primers used for serially deleted *ventx3.2* reporter gene constructs

Primer	Primer name	Sequence (5′-3′)
Upstream primer	V.x3.2(-1188)-Forward	AGTGTTTGCCCTGACCCTTAG
	V.x3.2(-1014)-Forward	AGCTAGTTGCCATGGTGCTTGC
	V.x3.2(-642)-Forward	TAGTGCCGTCCAGAAGATCAGA
	V.x3.2(-445)-Forward	AGAGGCCAGACATGATATGATC
	V.x3.2(-243)-Forward	AGAGATGAACTTGGTGCCAAG
	V.x3.2(-197)-Forward	AGGGATTAGCCAGATATGACA
	V.x3.2(-167)-Forward	GAATGCAGATTAGTGTAACAC
	V.x3.2(-47)-Forward	TTCAGTTTGGGCAGTCAGTGA
		
Downstream primer	V.x3.2-Reverse	AAGGTCTCTTCTCCATAGTCC

### Ventx3.2 Promoter Constructs

The *ventx3.2(-1188)-luc* construct was utilized to create the *ventx3.2* promoter construct through sequential deletion (Table 1). The *Nhe*I and *Xho*I restriction enzymes were used upstream and downstream, respectively, to digest and subclone the polymerase chain reaction (PCR) product into the pGl3-basic plasmid (Table 1).

### Embryo Injection and Explant Culture

Oocytes were obtained by administering 500 units of human chorionic gonadotropin to female *X. laevis* (Sigma-Aldrich). The obtained eggs underwent in vitro fertilization, and embryos at the 1-cell stage were injected with DNA and messenger RNA (mRNA) at the animal pole, as described previously (Kumar et al., 2023). At the blastula stage (stage 8), the AC explants were dissected from the injected and non-injected embryos. Whole embryos (WEs) and dissected ACs were cultivated until stages 11 to 11.5 using 1× L-15 medium (Gibco/Thermo Fisher) and 30% Marc’s Modified Ringer’s solution, respectively.

### Reverse Transcription-PCR

At the 1-cell stage, *X. laevis* embryos were injected into the animal pole containing 0.5 ng/embryo Gsc mRNA and 1 ng/embryo Cdx1 mRNA. Subsequently, the ACs were isolated from injected and non-injected embryos and cultivated in 1× L-15 growth medium until they reached stages 11 to 11.5, as described previously (Kumar et al., 2019). Total RNA was extracted from WEs and ACs using the TRIzol reagent (Ambion) according to the manufacturer’s instructions. The extracted RNA samples were treated with DNase I to eliminate impurities originating from genomic DNA. Next, reverse transcription-PCR (RT-PCR) was performed using Superscript-IV (Invitrogen) and 1 µg total RNA per reaction according to the manufacturer’s guidelines. The thermal cycling protocol was as follows: 30 s at 95 °C, 30 s at 57 °C, 30 s at 72 °C, and 20 to 30 amplification cycles (Table 2).

**Table 2:** Primers used for RT-PCR amplification

Gene name	Sequence (5′-3′)	Annealing temperature (°C)	Cycles
*Ventx1.1*	Forward: CCTTCAGCATGGTTCAACAG	57	25
	Reverse: CATCCTTTCTCCTTGGCATCTCCT		
*Ventx 2.1*	Forward: CTACAGCACTAGCACTGACTCAGG	57	27
	Reverse: TTGGACTGCATGCTGGAATACAGG		
*Ventx3.2*	Forward: TTCCGACCAAAGCACTGGAG	57	27
	Reverse: TTGCATCTTGTGTTTGACGCT		
*Bmp4*	Forward: CATCATGATTCCTGGTAACCGA	57	25
	Reverse: CTCCATGCTGATATCGTGCAG		
*Cdx1*	Forward: AACCCAACACAGATCGCTTT	57	25
	Reverse: TTCTAATTCAAGGCGCTGGT		
*xBRA*	Forward: CACCCAGACTCACCCAACTT	57	25
	Reverse: TTCATTCTGGTATGCGGTCA		
*Gsc*	Forward: GAATGGGCCGTTGCTCTT	57	28
	Reverse: TGAACTGCTCCACAACACGA		
*Chordin*	Forward:TTAGAGAGGAGAGCAACTCGGGCAAT	57	28
	Reverse: GTGCTCCTGTTGCGAAACTCTACAGA		
*Odc*	Forward: GTCAATGATGGAGTGTATGGATC	55	26
	Reverse: TCCATTCCGCTCTCCTGAGCAC		

### Quantitative RT-PCR

Samples from all quantitative RT-PCR (RT-qPCR) and ChIP experiments were analyzed using the Biosystems StepOnePlus Real-Time PCR System with Q712-00 Taq Pro Universal SYBR qPCR Master mix (Vazyme). The results were standardized to those of ornithine decarboxylase, a ubiquitous gene in *Xenopus* embryos that is expressed throughout embryonic development. Graphs were generated using the threshold cycle (Ct) values, delta cycle thresholds, delta-delta cycle thresholds, and the relative fold-change expression method.

### Luciferase Assays

Serially deleted and mutated *ventx3.2(-1188)luc* constructs were injected with or without *Cdx1* and *gsc* mRNAs. Reporter experiments were then conducted as described previously (Umair et al., 2021). The reporter gene assay involved measuring the relative promoter activity using a luciferase assay kit according to the manufacturer’s instructions (Promega). At stages 11 to 11.5, the embryos were divided into 5 groups (*n* = 3 embryos per group). Then, 10 µL lysis buffer was added to each embryo. Reporter gene activity was assessed through an illuminometer (Berthold Technologies) using 10 µL of each embryo homogenate and 40 µL of luciferase substrate. Each experiment was conducted autonomously and in triplicate (at a minimum).

### Site-directed Mutagenesis

Site-directed mutagenesis was conducted using a mutagenesis kit (Muta-Direct, iNtRON Biotechnology) and specific primer oligonucleotides (Table 3) as previously described and according to the manufacturer’s instructions (Umair et al., 2021).

**Table 3:** Primers used for site-directed mutagenesis

Mutated region	Primer name	Sequence (5′-3′)
GRE1	V3.2(GRE1)-Forward	AGATATGACAGCCC**GG**TGAGAATGCAGATTA
	V3.2(GRE1)-Reverse	TAATCTGCATTCTCA**CC**GGGCTGTCATATCT
GRE2	V3.2(GRE2)-Forward	ATTTATAGTCAG**GG**AGAGATGAGACTG
	V3.2(GRE2)-Reverse	CAGTCTCATCTCT**CC**CTGACTATAAAT
CRE1	V3.2(CRE1)-Forward	AGGGTTTAAATA**CC**TGTGCAACCTCAG
	V3.2(CRE1)-Reverse	CTGAGGTTGCACA**GG**TATTTAAACCCT

### ChIP Assay

ChIP was performed as described previously (Kumar et al., 2023). Myc-Cdx1 (1 ng/embryo) and Flag-Gsc (0.5 ng/embryo) mRNAs were introduced at the single-cell stage. Approximately 100 injected embryos per sample were collected and handled using established techniques at stages 11 to 11.5. Chromatin was then subjected to immunoprecipitation using anti-myc (for Cdx1 injected samples) and anti-FLAG (for Gsc-injected samples) polyclonal antibodies (SC-805, Santa Cruz Biotechnology, USA) or normal mouse IgG (SC-2025, Santa Cruz Biotechnology) in the cell lysates. ChIP-PCR was conducted using the immunoprecipitated chromatin; specific primers targeting the Cdx1 response element (CRE) and Gsc response element (GRE) were used (Table 4).

**Table 4:** Primer used in ChIP-PCR

Primer name	Sequence (5′-3′)	Annealing temperature (°C)
V.x3.2-197_ChIP_Forward	AGGGATTAGCCAGATATGACA	57
V.x3.2-197_ChIP_Forward	AAGGTCTCTTCTCCATAGTCC	57
V.x3.2_Chip_Negative_F	TGCAAGGGTTGAAGCTTTCT	57
V.x3.2_Chip_Negative_R	GAGCCAAAGTTTGCTGAAGG	57

### ChIP-Sequencing Analysis

In total, 600 to 700 embryos were injected and collected at stage 11 after the injection of gsc mRNA (0.5 ng/embryo) at the 1-cell stage. The ChIP assay was performed as described previously (Blythe et al., 2009). The immunoprecipitated chromatin was sequenced by Macrogen, and raw data (short reads) were obtained in the Fast Adaptive Shrinkage Threshold Algorithm (FASTA) format. Visualization was performed using the online data analysis tool Galaxy (https://usegalaxy.org). Gsc coverage was plotted together with the display of Model based analysis of ChIP-Seq (MACS) call peak data for the *ventx3.2* promoter region.

### In Silico Analysis

Ventx1.1, Ventx1.2, Ventx2.1, Ventx2.2, Ventx3.1, and Ventx3.2 protein sequences were obtained from “Xenbase” (https://xenbase.org) in the FASTA format. The “Pairwise alignment” tool in “Clustal Omega” was used for a comparative analysis of amino acid sequences. The online tool “SMART-PROTEIN Analysis” was utilized to select the homeodomains (HDs) of Ventx proteins.

### Morpholino Oligos (MOs)

Gene Tools LLC provided the antisense MOs targeting *X. laevis Cdx1* and *Gsc*, with the following sequences:

*Cdx1*: 5′-CCACGTACATCTTTGCCAAGAAATC-3′;

*Gsc*: 5′-GCTGAACATGCCAGAAGGCATCACC-3′.

Gsc morpholino (Gsc-MO) was prepared as described previously (Sander et al., 2007). MOs were reconstituted to a concentration of 1 mM in sterile water. Before being microinjected, the MOs were heated at 65 °C and then injected (as indicated) in the ventral and dorsal blastomeres at the 4-cell stage.

### Statistical Analysis

The obtained data were analyzed using GraphPad Prism (version 9.0, GraphPad Software) employing either a standard 1-way analysis of variance or an unpaired 2-tailed Student’s t-test (GraphPad Prism). “n.s.” represents nonsignificant results; *P < .1, **P < .01, ***P < .001, and ****P < .0001 were considered statistically significant.

## RESULTS

### Ventx3.2 is a Zygotic Gene Co-expressed With Ventx Family Members

**Fig. 1:**
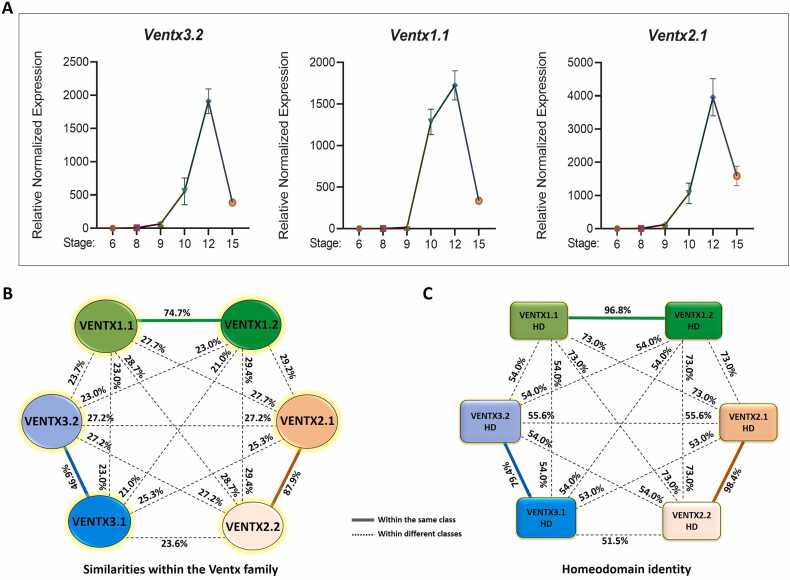
Zygotic transcription and protein identity comparison of ventx family members*.* (A) Temporal expression patterns of *ventx* genes in whole embryos, as determined using RT-qPCR. (B) Protein sequences downloaded from “Xenbase,” compared for identity using Clustal omega, and schematically drawn. (C) Amino acid sequences of conserved homeodomains (HD) selected and compared for identity using the “Clustal omega Pairwise alignment tool” and schematically drawn. Solid color lines depict identity within the same class, whereas dotted lines indicate identity within different classes of the ventx family.

Similar to other Ventx family members, *Ventx3.2* had discernible transcript levels near the mid-blastula transition (stages 8-9; Fig. 1A). *Ventx3.2* transcript levels increased shortly after stage 9 and peaked during the early to mid-gastrula stages (stages 10-12). Then, they sharply decreased at neurula stage 15, similar to those of other *ventx* members (Fig. 1A). The expression pattern of *ventx3.2* was comparable to that of well-known ventx members, such as *ventx1.1* and *ventx2.1*. This similarity suggests that these genes are likely regulated in a similar manner and have shared functions throughout the early development of *Xenopus*. As evolutionarily related proteins often share conserved regulatory elements, such as transcription factor (TF)-binding sites and enhancers, we used VENTX to determine the similarities in amino acid sequences. The amino acid sequence of Ventx3.2 was 46.9%, 23.7%, and 27.2% identical to that of Ventx3.1, Ventx1.1, and Ventx2.1, respectively (Fig. 1B). Next, the homeobox domain (HD) of Ventx3.2 was examined to determine its identity. The HD of ventx3.2 was 54%, 55.6%, and 79.4% identical to that of ventx1.1, ventx2.1, and ventx3.1, respectively (Fig. 1C). These findings indicate that ventx3.2 is a divergent ventx family member exhibiting the least resemblance to ventx1.1 and ventx1.2, followed by ventx2.2 and ventx2.1. The slightly reduced divergence with Ventx2 suggests a common evolution for ventx3 and ventx2. Despite its divergence from other Ventx members, Ventx3.2 exhibits an early expression pattern and function that resembles the role of BMP4 in limiting the Spemann organizer.

### Ventx3.2 Transcription is Upregulated by Cdx1 and Downregulated by Gsc

**Fig. 2:**
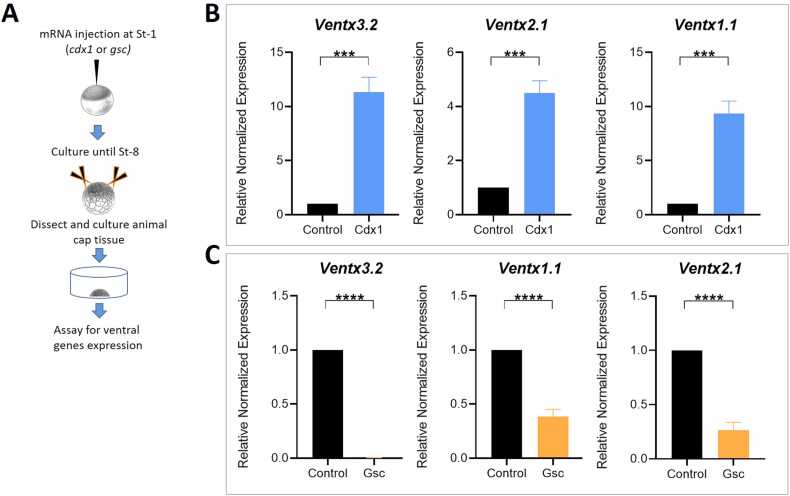
Expression of Cdx1 and Gsc ectopically modulates *ventx3.2* transcription differentially. (A) A schematic of the methodology for the ventral gene expression assay. (B) RT-qPCR results for ventral-specific *ventx1.1*, *ventx2.1*, and *ventx3.2* in samples injected with Cdx1. (C) RT-qPCR results for the above-mentioned genes in samples injected with Gsc. Fold enrichment was utilized for RT-qPCR analysis, and experiments were performed thrice. ***P ≤ .001 and ****P ≤ .0001 are the significant values assigned in (B and C). Gsc, goosecoid; Cdx1, caudal type homeobox 1.

In this study, the temporal expression patterns of both *Cdx1* and *Gsc* overlapped with those of *ventx3.2*. (Supplementary Fig. 1A and B). Thus, it was hypothesized that Cdx1 and Gsc would be present in the ventral and dorsal areas of the embryo, respectively, to precisely regulate the ventral-specific gene *ventx3.2*. We investigated the effects of Cdx1 and Gsc on the endogenous expression of ventx3.2 by administering *cdx1* and *gsc* mRNA through injection (Fig. 2A). The injection of Cdx1 mRNA resulted in the activation of ventral genes, such as *ventx3.2*, *ventx1.1*, and *ventx2.1*, in the AC. The result was confirmed using RT-qPCR and RT-PCR analyses (Fig. 2B and Supplementary Fig. 2A). Conversely, the endogenous expression of ventral genes, such as *ventx3.2*, in the AC was diminished by *Gsc* mRNA (Fig. 2C and Supplementary Fig. 2B). These findings imply that Cdx1 and Gsc exert opposite effects on *ventx3.2* expression in *X. laevis*.

### Gsc and Cdx1 Response Elements are Located Within the Proximal Region of the ventx3.2 Promoter

**Fig. 3:**
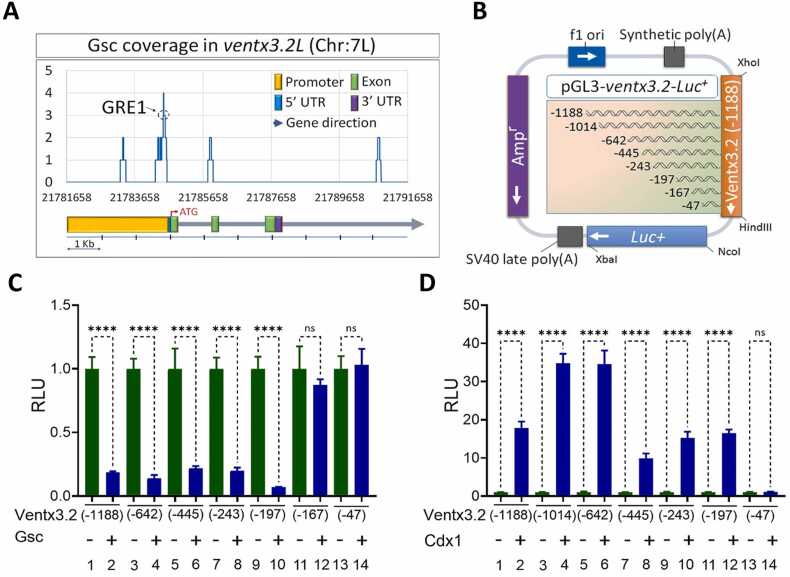
Chromatin immunoprecipitation-sequencing and reporter gene assay mapping of the response elements of Gsc and Cdx1 within the *ventx3.2* promoter region. (A) A plot of 3Flag-Gsc coverage within the *ventx3.2* promoter region. (B) A map of serially deleted *ventx3.2* promoter constructs. (C and D) Relative promoter activities of serially deleted constructs of *ventx3.2* promoters injected with or without *Gsc* (C) and *Cdx1* (D) mRNA at the 1-cell stage. (C, D) ****P ≤ .00001 indicates statistical significance; ns indicates nonsignificant values. Gsc, goosecoid; Cdx1, caudal type homeobox 1; RLU, relative luciferase unit.

ChIP-sequencing analysis results showed that Gsc was bound to the upstream promoter region of *ventx3.2*. Peak calling, followed by coverage plot analysis, helped in the identification and mapping of probable Gsc-binding sites in the promoter region of *ventx3.2* (Fig. 3A). Examination of the ChIP coverage area of Gsc revealed the presence of 2 putative GREs in the region upstream of the *ventx3.2* coding sequence. To identify the actively functioning *GRE*, we created luciferase constructs that included *ventx3.2* promoter regions with sequential deletions (Fig. 3B). Gsc reduced the promoter activity of *ventx3.2(-1188)luc* by 0.2-fold (Fig. 3C, bars 1-2). To delineate the functional GRE within the *ventx3.2(-1188)* promoter, we employed a series of deleted *ventx3.2* constructs. Coinjecting *Gsc* mRNAs with ventx3.2-deleted constructs considerably reduced the promoter activity from *-*1188 to *-*197 bp (Fig. 3C, bars 3-10). However, deletion of the constructs at -167 and -47 bp did not cause any significant reduction in promoter activity (Fig. 3C, bars 11-14). These data suggest that the putative active GRE could be found within -197 to -47 bp of the *ventx3.2* promoter.

Next, we investigated the effect of Cdx1 on the *ventx3.2(-1188)* promoter. Consistent with RT-qPCR results, simultaneous expression of *Cdx1* mRNA with the *ventx3.2(-1188)* promoter markedly increased the activity of the *ventx3.2(-1188)* to *ventx3.2(-197)* promoter, reaching up to 35-fold (Fig. 3D). Examination of the deleted promoter constructs of *ventx3.2* revealed that Cdx1 did not impact the activity of the *ventx3.2(-47)* bp promoter construct (Fig. 3D, bars 13-14). This result suggests that the CRE is located upstream of the -47 bp region of the *ventx3.2* promoter.

### Site-directed Mutagenesis of GRE and CRE in the *ventx3.2* Promoter Uniquely Eliminates Gsc- and Cdx1-Mediated Transcription

**Fig. 4:**
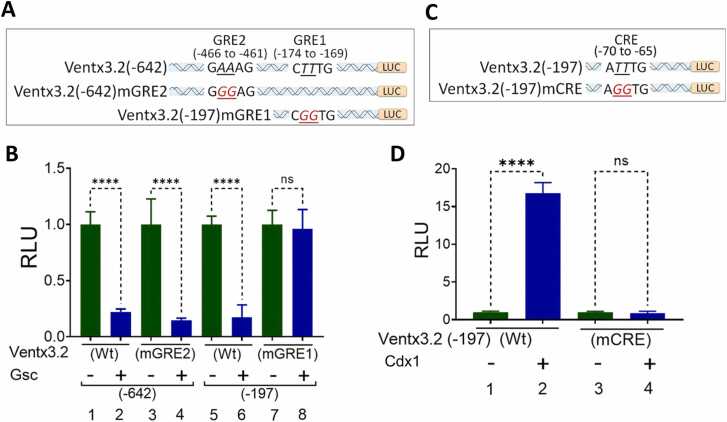
Site-specific mutations of Gsc response element (GRE) and Cdx1 response element (CRE) within the ventx3.2 promoter eliminates Gsc and Cdx1 activities. Systemic representation of mutated GREs (A) and CREs (C) within the *ventx3.2* promoter (targeted nucleotides are shown in italics and underlined). (B) Relative luciferase activity at embryonic stage 11 for *ventx3.2(-642)*, *ventx3.2(-642)mGRE2*, *ventx3.2(-197)*, and *ventx3.2(-197)mGRE1* (40 pg/embryo) without or with *Gsc* mRNA (500 pg/embryo) injected at the 1-cell stage. (D) Relative luciferase activity for *ventx3.2(-197)mCRE* and *ventx3.2(-197)mCRE* injected at 40 pg/embryo with or without *Cdx1* mRNA (1 ng/embryo) and harvested at stage 11. (B and D) ****P ≤ .00001 indicates statistical significance; ns indicates nonsignificant values. Gsc, goosecoid; Cdx1, caudal type homeobox 1; RLU, relative luciferase unit.

Caudal type TFs have selectivity for a conserved DNA sequence with the consensus sequence 5′-ATTT-3′ (Hwang et al., 2002a, Kumar et al., 2019). A thorough examination of the *ventx3.2(-1188)* promoter sequence revealed a single potential CRE (*ATTTG*) between positions -70 and -65 bp. We also identified 2 GREs ,GRE1 and GRE2 between positions -174 to -169 (*CTTTG*) and -466 to -461 (*GAAAG*), respectively (Fig. 4A and C). To investigate whether the GREs and CRE function as response elements for Gsc and Cdx1, we introduced a point mutation through site-directed mutagenesis by altering the 2 conserved nucleotides within the response elements (Fig. 4A and C). The reporter gene assay was conducted using the mutant *ventx3.2(-197)mGRE1* and *ventx3.2(-642)mGRE2* and wild-type versions of the promoters, both with and without Gsc, at stage 11.

The reduction in *ventx3.2(-197)* and *ventx3.2(-642)* promoter activities caused by Gsc was completely eliminated in the mutant *ventx3.2(-197)mGRE1* but not in *ventx3.2(-642)mGRE2* (Fig. 4B). These findings suggest that the functionally active Gsc consensus sequence is located in the -174 to -169 bp area within the *ventx3.2(-197)* promoter region. This area is located where Gsc binds to *ventx3.2* and inhibits its transcription. Next, we analyzed the mutant promoter variant *ventx3.2(-197)mCRE* and the wild-type *ventx3.2(-197)* with or without Cdx1 (Fig. 4D). Activation of the *ventx3.2* promoter activity by Cdx1 was completely abolished in the mutant variant of *ventx3.2(-197)mCRE* (Fig. 4D). This indicates that the functionally active CRE is situated within the -70 to -65 bp region of the *ventx3.2* promoter. Both datasets indicate that the proximal region of the *ventx3.2* promoter contains functional *GRE* and *CRE* consensus sequences that regulate its activity.

### Gsc and Cdx1 Occupy GRE1and CRE to Differentially Modulate *ventx3.2* Transcription

**Fig. 5:**
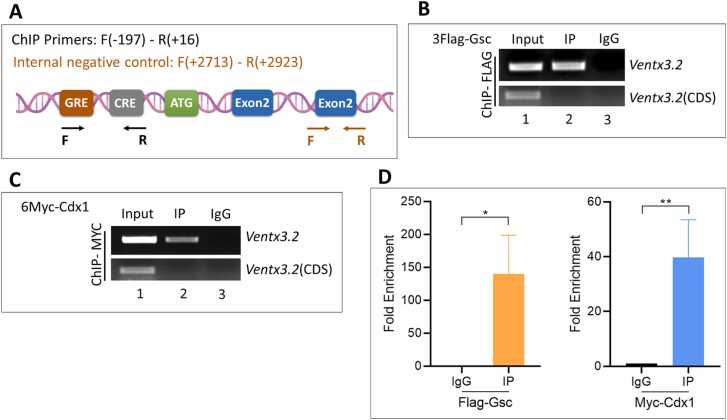
Gsc and Cdx1 bind to the *ventx3.2* proximal promoter region. (A) Schematic representation of common ChIP-PCR primers F (forward) and R (reverse) as well as the location of GRE and CRE in the ventx3.2 promoter. (B, C) ChIP-PCR results showing the interaction between Flag-Gsc and GRE and between Myc-Cdx1 and CRE. Common ChIP primers (containing both GRE and CRE) were used for amplification, while *ventx3.2* CDS (exon 3) primers served as the negative control for both. (D) ChIP-qPCR results showing the occupancy of Myc-Cdx1 on CRE and Flag-Gsc on GRE. Fold enrichment was utilized to normalize ChIP-qPCR readings. *P ≤ .1 and **P ≤ .01 indicate statistical significance. Gsc, goosecoid; Cdx1, caudal type homeobox 1; CRE, Cdx1 response element; GRE, Gsc response element; ChIP-PCR, chromatin immunoprecipitation-polymerase chain reaction; IgG, immunoglobulin G; IP, immuno precipitated; CDS, coding sequence

Promoter regions carrying GRE1 and CRE mutations led to a significant reduction in the baseline activity of the deleted *ventx3.2(-197)* promoter (Fig. 4). This suggests that the response elements may be occupied by the transcriptional repressor Gsc and activator Cdx1. Hence, we examined the direct interaction between TFs Gsc and Cdx1 with the proximal region of the endogenous *ventx3.2* promoter. Flag-Gsc- and Myc-Cdx1-injected embryos at the gastrula stage were subjected to direct-binding ChIP to confirm their interaction. Following Flag- and Myc-antibody immunoprecipitation against injected Flag-Gsc and Myc-Cdx1 with total chromatin, ChIP-PCR, and ChIP-qPCR were performed. Gsc and Cdx1 are directly bound to the proximal region of the endogenous *ventx3.2* promoter (Fig. 5B and C, lane 2). ChIP-qPCR results indicated that Gsc predominantly occupied the GRE, whereas Cdx1 occupied the CRE (Fig. 5D). These findings suggest that the *ventx3.2* promoter has specific regions called *cis*-acting GRE (-174 to -169 bp) and CRE (-70 to -65 bp). Gsc directly binds to the GRE region and downregulates *ventx3.2* transcription, whereas Cdx1 binds to the CRE region to upregulate *ventx3.2* transcription.

### Cdx1 Knockdown Selectively Restricts *ventx3.2* Expression in the VMZ, Whereas Gsc Depletion Promotes it in the Dorsal Marginal Zone

**Fig. 6:**
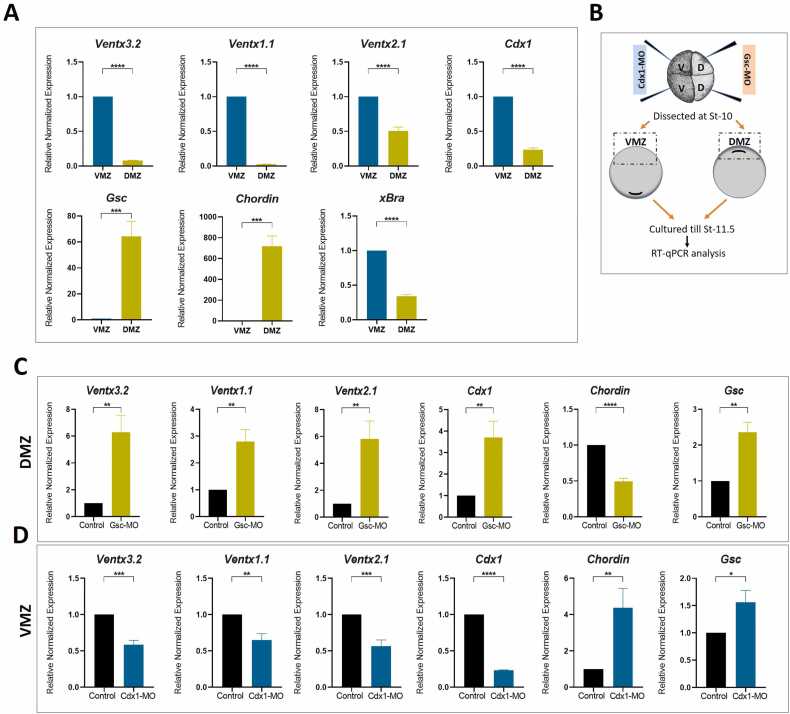
Gsc knockdown induces *ventx3.2* transcription, whereas Cdx1 knockdown reduces it in the DMZ and VMZ, respectively. (A) Endogenous ventx3.2 expression in the VMZ in *Xenopus* embryos, with *xBra* as the pan-mesodermal marker. (B) Schematic representation of the experimental design for VMZ and DMZ analyses. (C) Gsc knockdown at dorsal blastomeres at the 4-cell stage induces *ventx3.2* transcription in the DMZ during gastrulation. (D) Cdx1 knockdown in ventral blastomeres at the 4-cell stage reduces *ventx3.2* transcription in the VMZ. *P ≤ .1, **P ≤ .01, ***P ≤ .001, and ****P ≤ .0001 are considered statistically significant. Gsc, goosecoid; VMZ, ventral marginal zone; DMZ, dorsal marginal zone; Cdx1, caudal type homeobox 1; Xbra, *Xenopus* brachyury.

In this study, we postulated that Gsc limits *ventx3.2* transcription in the dorsal marginal zone (DMZ), whereas Cdx1 positively regulates it in the VMZ. To verify our hypothesis, we first examined the endogenous expression of *ventx3.2* in VMZ and DMZ explants. At the start of gastrulation, the VMZ and DMZ were extracted separately and cultivated until stages 11 to 11.5 for RT-qPCR analysis. *Ventx3.2* expression was considerably greater in the VMZ explants than in the DMZ explants, which is consistent with our hypothesis (Fig. 6A, bar graph 1). The expression of *Gsc* and Chordin prevailed in the DMZ; in contrast, that of ventral members, such as *ventx1.1, ventx2.1*, and *Cdx1*, was predominant in the VMZ (Fig. 6A). This spatial expression pattern suggests the presence of different regions of dominance that contribute to the overall regulation of embryonic development in *Xenopus*.

To determine whether the reduction in Gsc and Cdx1 expression in the DMZ and VMZ, respectively, facilitates or impedes *ventx3.2* transcription, we employed a loss-of-function strategy (Fig. 6B). We designed a Cdx1 morpholino and used a previously reported Gsc-MO targeting *cdx1* and *gsc* in *X. laevis* (Supplementary Fig. 3A). To observe the impact of the loss of function of each gene, we first adjusted the concentration for each morpholino. Headless phenotypes were produced when 70 ng Gsc-MO was injected into each dorsal blastomere at the 4-cell stage (Supplementary Fig. 3B). The expression of *ventx3.2* in the Gsc-depleted DMZ was increased by 6-fold (Fig. 6C, bar graph 1). The expression of ventral genes, such as *ventx1.1, ventx2.1 and cdx1* was also significantly elevated upon Gsc inhibition in the DMZ (Fig. 6C, bar graphs 2, to 4), whereas that of the dorsal-specific gene chordin was reduced by 2-fold (Fig. 6C, bar graph 5). These results indicate that Gsc negatively regulates ventral genes expression (Fig. 6C).

To assess the potential for Cdx1-mediated upregulation of ventx3.2, we doubly knocked down both Gsc and Cdx1 in the DMZ (Supplementary Fig. 4A). Double knockdown resulted in an increase in the expression of ventx3.2 but compared to Gsc depletion alone it decreased by approximately 6-fold to 2-fold (Fig. 6C and Supplementary Fig. 4B, bar graph 1). This suggests that Cdx1 may play a positive role in the regulation of ventx3.2 expression. The expression of other ventral genes, including *ventx1.1*, *ventx2.1*, and *bmp4*, showed an identical pattern (Supplementary Fig. 4). These results indicate that Cdx1 might positively regulate the expression of ventral genes, including *ventx3.2*.

We investigated the impact of Cdx1 on the regulation of ventx3.2 in the VMZ, as the expression of Bmp and Cdx1 is particularly prominent in this region. We injected Cdx1 MO (38 ng) into each ventral blastomere at the 4-cell stage. The inhibition of Cdx1 expression in the VMZ led to a reduction in the expression of *ventx3.2* and other markers associated with the ventral region (Fig. 6D). Moreover, the reduction of Cdx1 expression resulted in the activation of organizer genes (*chordin* and *gsc*) in the VMZ (Fig. 6D, bar graphs 5 and 6). The modulation of Cdx1 expression in the VMZ using its morpholino oligo (MO) resulted in the development of embryos with shortened or curved axis and phenotypes characterized by posterior truncation (Supplementary Figs. 3C and 4E).

The reduced expression of *ventx3.2* compared to that of other ventral genes implies that the regulation of the expression of *ventx* family members in the VMZ requires normal Cdx1 levels. Furthermore, the additional Cdx1 knockdown in the DMZ with Gsc resulted in the induction of *ventx3.2* expression. Therefore, we investigated whether Gsc knockdown can restore *ventx3.2* expression in the Cdx-depleted VMZ. The VMZ still showed reduced expression of *ventx3.2* (Supplementary Fig. 4C). We observed a decrease in the induction of *gsc* and *chordin* expression in the VMZ upon comparing the Cdx1/Gsc-depleted VMZ with the Cdx1-depleted VMZ (Supplementary Fig. 4C, bar graphs 6 and 7). These findings suggest that ventx3.2 expression particularly requires a minimal amount of Cdx1 in the VMZ. Overall, we can conclude that Gsc from the dorsal region is essential for limiting the expression of *ventx3.2*. However, Cdx1 is crucial for the positive expression of *ventx3.2* in the ventral region.

## DISCUSSION

In this study, we investigated the regulatory mechanisms underlying *ventx3.2* transcription during gastrulation in two embryonic regions. We focused on Ventx3.2, a repressive ventral-specific HD TF that is poorly studied. *Ventx3.2* transcription began zygotically at blastulation (stages 8/9) and peaked at mid-gastrulation (stage 12) (Fig. 1A). The ability of overexpressed Ventx3.2 to ventralize *Xenopus* embryos indicates its crucial role in specifying ventral cell fates during development. Its loss-of-function phenotype causing dorsalization further underscores its importance in pathways critical for DV patterning. The complexity of developmental biology is highlighted by the fact that Ventx3.2 has the least sequence identity with ventx family members (Fig. 1B) but still exerts crucial effects. This uniqueness could hint at specialized functions or regulatory mechanisms that distinguish Ventx3.2 from its other ventx counterparts.

Ventx3.2 restores BMP4 signaling and restricts dorsal mesoderm expansion (Shapira et al., 2000, Shapira et al., 1999). Moreover, it directly suppresses the expression of organizer and dorsal mesoderm genes, such as *Gsc* and *Otx2*, to maintain ventral mesoderm identity (Shapira et al., 2000). Although ventx3.2 is critical for normal development, the molecular mechanism that regulates its expression remains unknown. In the present study, both gain- and loss-of-function approaches were used to investigate the mechanism underlying *ventx3.2* regulation in *Xenopus*. Although the effects of ventx3.2 on Gsc and Otx2 in the ventral mesoderm have been identified, how Gsc counteracts it in the dorsal mesoderm remains unknown. Studies on *Xenopus* have shown that Gsc overexpression inhibits BMP4 signaling and its downstream targets, resulting in the development of dorsalized embryos (Niehrs, 2001, Niehrs et al., 1993, Niehrs et al., 1994).

The integration of BMP and FGF signaling is crucial for embryonic patterning (Kumar et al., 2021a, Kumar et al., 2021b). Cdx1, a transcriptional activator dependent on FGF, exerts a positive regulatory effect on the expression of *ventx1.1* and *ventx2.1*, which are target genes of BMP4 during the embryonic development of *Xenopus* (Keenan et al., 2006, Kumar et al., 2019, Pillemer et al., 1998). However, whether ventx3.2 is positively regulated by Cdx1 remains unknown. Therefore, it is hypothesized that Gsc and Cdx1 may distinctly modulate the transcriptional regulation of *ventx3.2* expression.

### Gsc Inhibits *ventx3.2* Transcription in the DMZ to Maintain Dorsal Destiny

We used Gsc from the dorsal mesoderm to examine its inhibitory effect on *ventx3.2* transcription for the following reasons: First, Gsc stimulates the development of the dorsal mesoderm or organizer, which is crucial for the formation of embryonic patterns (Cho et al., 1991, Steinbeisser et al., 1993, Yao and Kessler, 2001). Second, Gsc represses the expression of genes that prevent the differentiation of the organizer or dorsal mesoderm (Christian and Moon, 1993, Steinbeisser et al., 1995). The antagonistic relationship between the *Gsc* and *Ventx* genes during embryonic patterning in *Xenopus* has been demonstrated (Sander et al., 2007, Lee et al., 2011). Gsc can counteract the ventralizing effect by repressing the expression of genes that are exclusive to the ventral region (Sander et al., 2007). However, the precise molecular mechanisms through which Gsc restricts the expression of ventx family members in the DMZ remain unclear.

Shapira et al. (1999) established a temporal window of competence between ventx3.2 and Gsc. As Gsc is expressed on the dorsal side of *Xenopus* early gastrulae, the absence of *Ventx3.2* transcription in that area may be attributed to Gsc. Evidence for this phenomenon was observed through the examination of the temporal expression patterns of both genes, as demonstrated by the intersection of Gsc expression with ventx3.2 expression (Fig. 1A and Supplementary Fig. 1B). Second, ectopic expression of *Gsc* mRNA considerably reduced the expression of endogenous *ventx1.1*, *ventx2.1*, and *ventx3.2* in the AC explants (Fig. 2B).

Gsc exhibits substantial repressor activity by directly binding to *cis*-acting response regions of the target genes, thereby inhibiting transcription (Cho et al., 1991, Latinkic and Smith, 1999, Ulmer et al., 2017). Previous research has demonstrated that Gsc significantly reduces *ventx1.1* promoter activity while having no effect on *ventx2.1* promoter (Lee et al., 2011). In this study, our first objective was to determine whether Gsc affects the promoter activity of *ventx3.2.* We used a *ventx3.2* reporter to investigate the potential direct effects of Gsc on ventx3.2. Here, Gsc overexpression reduced the *ventx3.2(-1188)luc* reporter gene expression in WEs (Fig. 3C). As the *Xenopus* genome has been successfully sequenced (Session et al., 2016), Gsc-based genome-wide ChIP-sequencing has been performed during *Xenopus gastrulation* (Umair et al., 2021). Our genome-wide ChIP-sequencing results revealed Gsc-binding sites in the 5′ flanking region of the *ventx3.2* promoter (from -466 to -169 bps) (Fig. 3A). The generation of several deleted promoter constructs, together with site-directed mutagenesis, revealed the presence of conserved *cis*-acting elements (GRE; GAAAG) in the promoter region. Point mutations were created to examine the functions of GRE1 and GRE2. We found that Gsc lost its repressor activity in the *ventx3.2(-197)mGRE1* reporter gene. The binding site of Gsc on the ventx3.2 promoter was further confirmed through ChIP-PCR (Fig. 5B). These findings indicate that Gsc may function independently as a repressor of ventx3.2 transcription. GRE1 (-174 to -169 bp) was identified in the ventx3.2 promoter in the present study.

Spatial expression patterns of *ventx3.2* transcription in the VMZ and DMZ aid in determining the spatial window wherein its expression is differentially regulated. Loss of function studies were conducted to demonstrate the inhibitory effect of Gsc in the DMZ system. Gsc knockdown affects head development and axis patterning, in addition to increasing ventx1.1 and ventx2.1 expression (Sander et al., 2007). We reconfirmed Gsc knockdown by generating headless phenotypes (Supplementary Fig. 3B and Fig. 4D). Gsc knockdown resulted in elevated *ventx3.2* expression, thus validating the negative effect of Gsc on *ventx3.2* in the DMZ (Fig. 6C).

The expression of Cdx1 was significantly increased in the DMZ when Gsc expression was blocked, as shown in Figure 6C. Cdx1 can enhance the expression of ventral genes (Keenan et al., 2006, Pillemer et al., 1998). Thus, we used a double knockdown approach to investigate whether this increased Cdx1 expression has an impact on the expression of ventx3.2 in the DMZ (Supplementary Fig. 4A). Cdx1 had a partially positive impact on the upregulation of ventx3.2 in the Gsc-depleted DMZ. Despite the Gsc-induced inhibition of Cdx1 expression, *ventx3.2* expression remained high in the DMZ (Supplementary Fig. 4B). Loss-of-function experiments confirmed that Gsc acts as a repressor for ventx3.2. Consequently, the removal of Gsc activates ventx3.2 transcription in the DMZ. By integrating both gain- and loss-of-function experiments, our study defines the regulatory mechanism through which Gsc suppresses *ventx3.2* transcription in the DMZ to maintain the dorsal identity.

### Cdx1 Activates *ventx3.2* Transcription in the Ventral Mesoderm During Embryonic Patterning

Among the 3 caudal genes in *Xenopus*, *CDX1* most obviously displays a dorsoventrally regulated pattern of expression, which indicates its potential function within the DV patterning network (Pillemer et al., 1998). Cdx1 activates BMP4 signaling and can emit a signal that causes ventralization during the early stages of gastrulation (Pillemer et al., 1998). We selected Cdx1 as an activator of ventx3.2 based on the following reasons: The postulation that *Cdx1* may serve as a ventral gene was first supported by the observation that the ventrolateral pattern of Cdx1 expression is comparable to the pattern of Bmp4 expression along the marginal zone during early gastrulation (Dosch et al., 1997, Fainsod et al., 1994, Pillemer et al., 1998). Second, Cdx1 overexpression leads to the dorsal enlargement of the xvent1 and xvent2/vox expression domains (Pillemer et al., 1998). Additionally, the ectopic expression of *Cdx1* mRNA significantly induces the transcription of *ventx1.1* and *ventx2.1* during gastrulation (Kumar et al., 2019, Pillemer et al., 1998), suggesting the essential regulatory function of Cdx1 in the transcription of ventx family members in *Xenopus*.

In the present study, the temporal expression patterns of *ventx3.2* and *Cdx1* overlapped during gastrulation (Fig. 1A and Supplementary Fig. 1A). The Gsc, Xnot-2, Otx-2, Xlim-1, and XFKH1 TFs are expressed only in the organizer during early gastrulation, and Cdx1 inhibits their expression in the ventral region (Blitz and Cho, 1995, Cho et al., 1991, Dirksen and Jamrich, 1992, Gont et al., 1993, Pannese et al., 1995, Pillemer et al., 1998, Taira et al., 1992). Furthermore, BMP4 is downregulated in the lateral marginal zone and VMZ when Cdx1 function is limited, which indicates that Cdx1 is crucial for BMP4 expression during gastrulation (Pillemer et al., 1998, Steinbeisser et al., 1995). This suggests that Cdx1 plays a crucial role in maintaining ventral signaling throughout gastrulation for both BMP4 and its target genes.

The present study elucidated the mechanisms through which Cdx1 activates *ventx3.2* at the transcription level in *Xenopus* embryos. To this end, we evaluated the expression of the endogenous *ventx3.2* gene and the reporter gene. Our findings demonstrated a significant increase in the expression of endogenous *ventx3.2* and reporter gene activity in response to Cdx1 expression. Cdx1 efficiently occupies the Ventx1.1 response element to increase the activity of the ventx1.1 promoter (Kumar et al., 2019). Therefore, we examined the CREs present in the *ventx3.2(-1188)* promoter. Serial deletions and site-directed mutagenesis on the *ventx3.2(-1188)* constructs suggest that CRE is a functional *cis*-acting element responsible for the Cdx1-mediated activation of ventx3.2 transcription.

Cdx1 can activate ventral genes without relying on Bmp4. However, its precise mechanism has not been elucidated (Pillemer et al., 1998). The presence of CRE within the ventx3.2 promoter indicates that Cdx1 independently activates ventx3.2 transcription. The synexpression patterns of ventx3.2 and Cdx1 in the VMZ are shown in Figure 6A. To assess the positive effect of Cdx1 on ventx3.2 transcription in VMZ, Cdx1 was depleted with MO. The depletion of Cdx1 in the VMZ suggested that it might serve as an activator, as it inhibited the normal expression of *ventx3.2*.

Consistent with our findings, previous studies have shown that antisense Cdx1 can stimulate the expression of organizer genes (Pillemer et al., 1998). Depletion of Cdx1 in the VMZ increased the expression of *gsc* and *chordin* (Fig. 6D) in the present study. Moreover, the normal expression of *ventx3.2* in the VMZ could not be restored by double silencing of Cdx1 and Gsc (Supplementary Fig. 4C), indicating that Cdx1 is essential for *ventx3.2* transcription in VMZ. Our adoption of a gain- and loss-of-function approach in this study demonstrated the positive interaction between Cdx1 and CRE in the *ventx3.2* promoter, which is crucial for maintaining the specification of the ventral lineage in *X. laevis.* A considerable decrease in BMP4 expression was observed in both the Gsc/Cdx1-depleted DMZ and Cdx1-depleted VMZ. In contrast, *ventx3.2* expression increased in the Gsc/Cdx1-depleted DMZ (Supplementary Fig. 4B) and was not eliminated in the Cdx1-depleted VMZ (Fig. 6D), indicating the involvement of an additional signaling pathway in its retention and positive regulation. However, further research is required to confirm the potential involvement of other BMPs or ventral signals in the transcriptional regulation of ventx3.2.

**Fig. 7:**
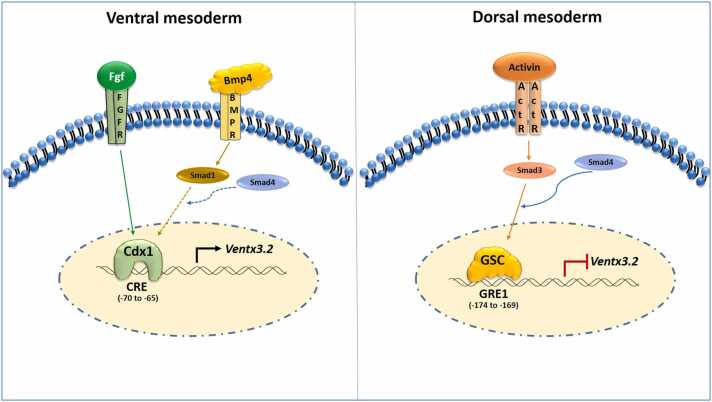
Model proposed for the regulation of *ventx3.2* transcription by Gsc and Cdx1. Schematic representation of the suggested molecular mechanism underlying *ventx3.2* transcriptional regulation in *Xenopus* gastrulae. Gsc, goosecoid; Cdx1, caudal type homeobox 1.

In conclusion, we demonstrate the rigorous regulation of *ventx3.2* transcription in the DMZ and VMZ during embryonic development. The transcription of *ventx3.2* in the VMZ is positively regulated by Cdx1 through CRE. In the DMZ, it is negatively regulated by Gsc through GRE. The proposed molecular mechanism by which Gsc and Cdx1 regulate ventx3.2 transcription and preserve ventral and dorsal mesodermal patterning is depicted in Figure 7. This study provides a novel understanding of the molecular process that underlies the dorso-ventral and anterior-posterior patterning of the vertebrate model system.

## Funding and support

This study was supported by the Basic Science Research Program of the National Research Foundation of Korea and funded by the Ministry of Education, Science, and Technology of Korea (grant numbers 2016R1D1A1B02008770, 2021M3H9A1097557, and 2021R1A4A1027355).

## Author contributions

R.S.G. designed and performed the experiments, contributed to data analysis and drafted the primary manuscript. V.K. designed and performed the experiments, contributed to data analysis, and edited the manuscript. U.L. and J.K. designed, supervised, and visualized all experiments and edited the final manuscript.

## Declaration of Competing Interests

The authors declare that they have no known competing financial interests or personal relationships that could have appeared to influence the work reported in this paper.
