# Supplementary material for: Cdx1 and Gsc distinctly regulate the transcription of BMP4 target gene ventx3.2 by directly binding to the proximal promoter region in Xenopus gastrulae
Source: Mol Cells. 2024 Mar 23;47(4):100058. doi: 10.1016/j.mocell.2024.100058 (PMC11031840; doi:10.1016/j.mocell.2024.100058)
Supplement: Supplementary file 1 — Supplementary material. [file mmc1.docx]

Cdx1 and Gsc distinctly regulate the transcription of Bmp4 target gene *ventx3.2* by directly binding to the proximal promoter region in *Xenopus* gastrulae^[[1]](#footnote-1)^

Ravi Shankar Goutam^a,d^, Vijay Kumar^a,b,d^, Unjoo Lee^c^***, and Jaebong Kim^a^*

^a^Department of Biochemistry, Institute of Cell Differentiation and Aging, College of Medicine, Hallym University, Chuncheon, Gangwon-Do 24252, Republic of Korea.

^b^Laboratory of Regenerative Medicine, College of Pharmacy, Ewha Womans University, Seoul, Republic of Korea.

^c^Department of Electrical Engineering, Hallym University, Chuncheon 24252, Korea.

^d^These authors contributed equally to this work.

***Address correspondence to:

Jaebong Kim

Fax: +82-33-244-8425;

Tel: +82-33-248-2544;

E-mail: [jbkim@hallym.ac.kr](mailto:jbkim@hallym.ac.kr) (JK), [ejlee@hallym.ac.kr](mailto:ejlee@hallym.ac.kr) (UL)


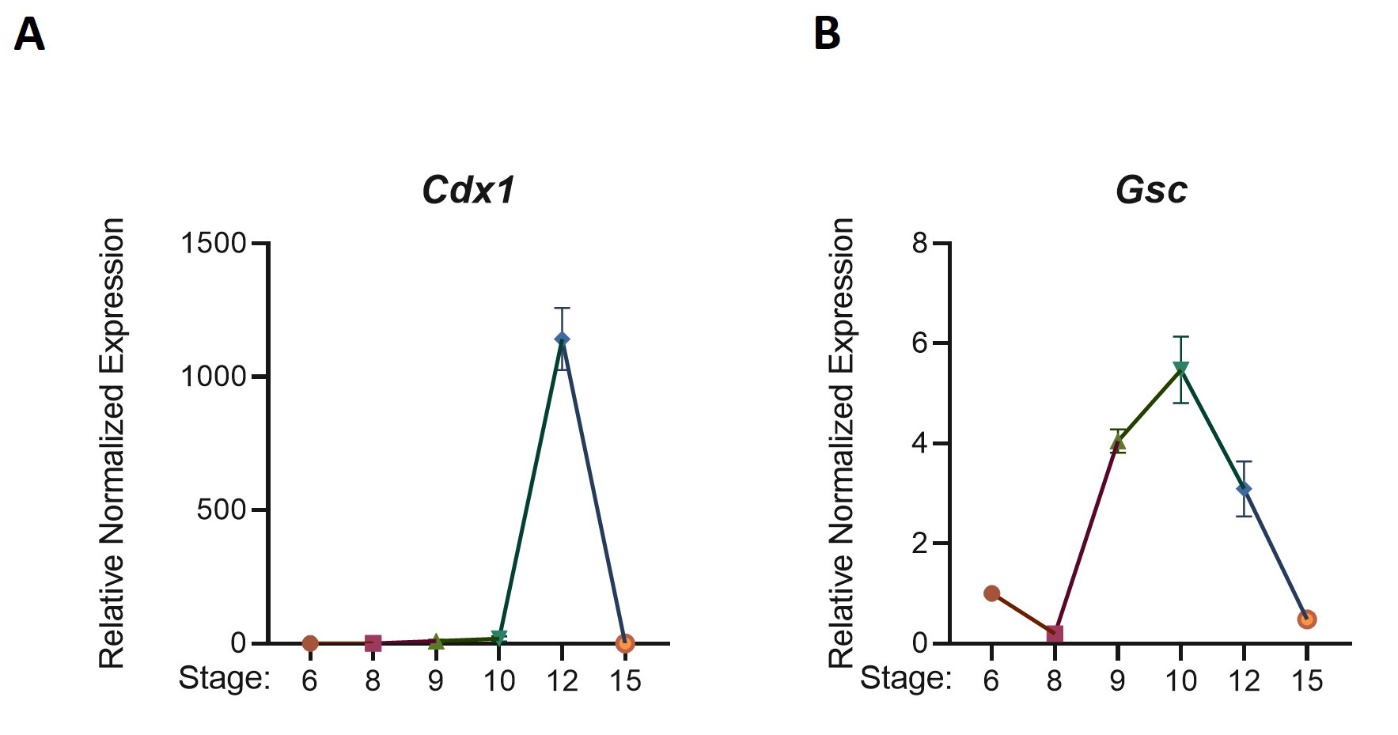


**Supplementary Fig. 1**. Stagewise expression patterns of Cdx1 and Gsc in *Xenopus laevis*. Whole embryos at different stages were harvested for expressional analysis. RT-qPCR was performed to analyze the stage-dependent expression of (A) *Cdx1* and (B) *Gsc*.


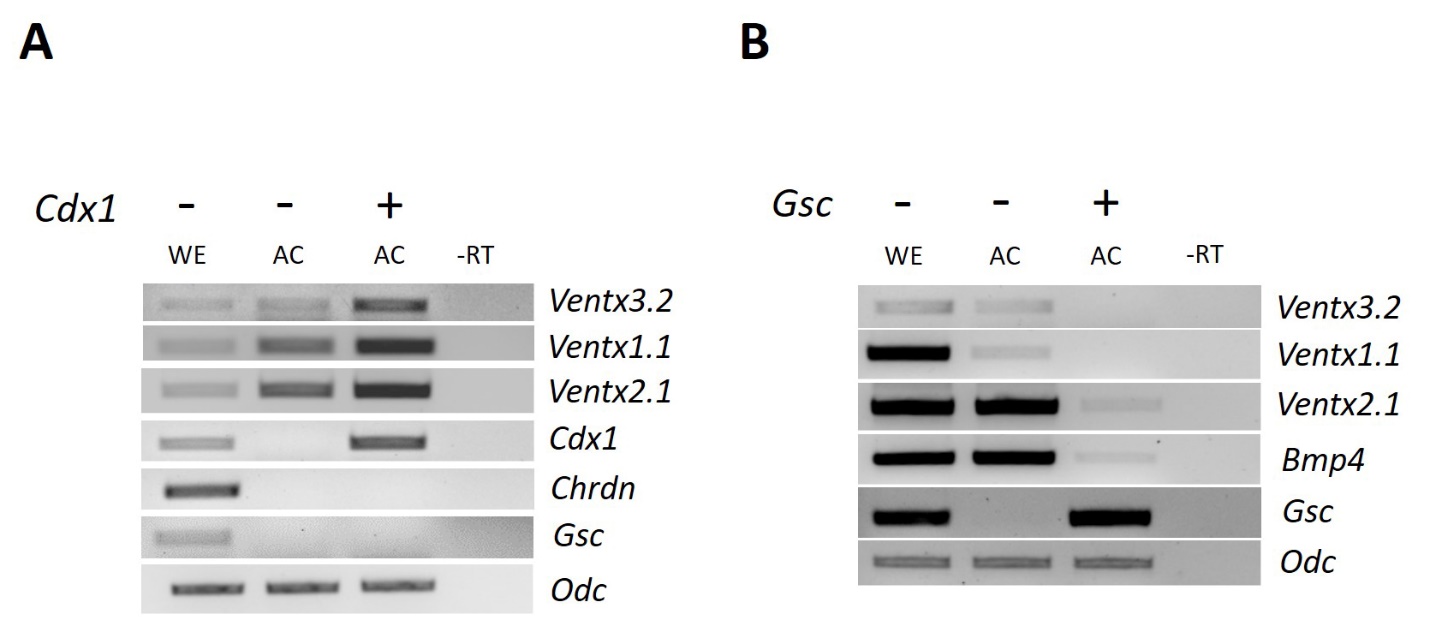


**Supplementary Fig. 2**. Ectopic expression of Cdx1 and Gsc in cap explants. (A) RT-PCR showings ventral and dorsal gene expression in the *Cdx1*-injected animal cap explants. (B) RT-PCR showings ventral and dorsal gene expression in *Gsc* -injected animal cap explants. Ornithrine de-carboxylase (Odc) was used as house-keeping gene.


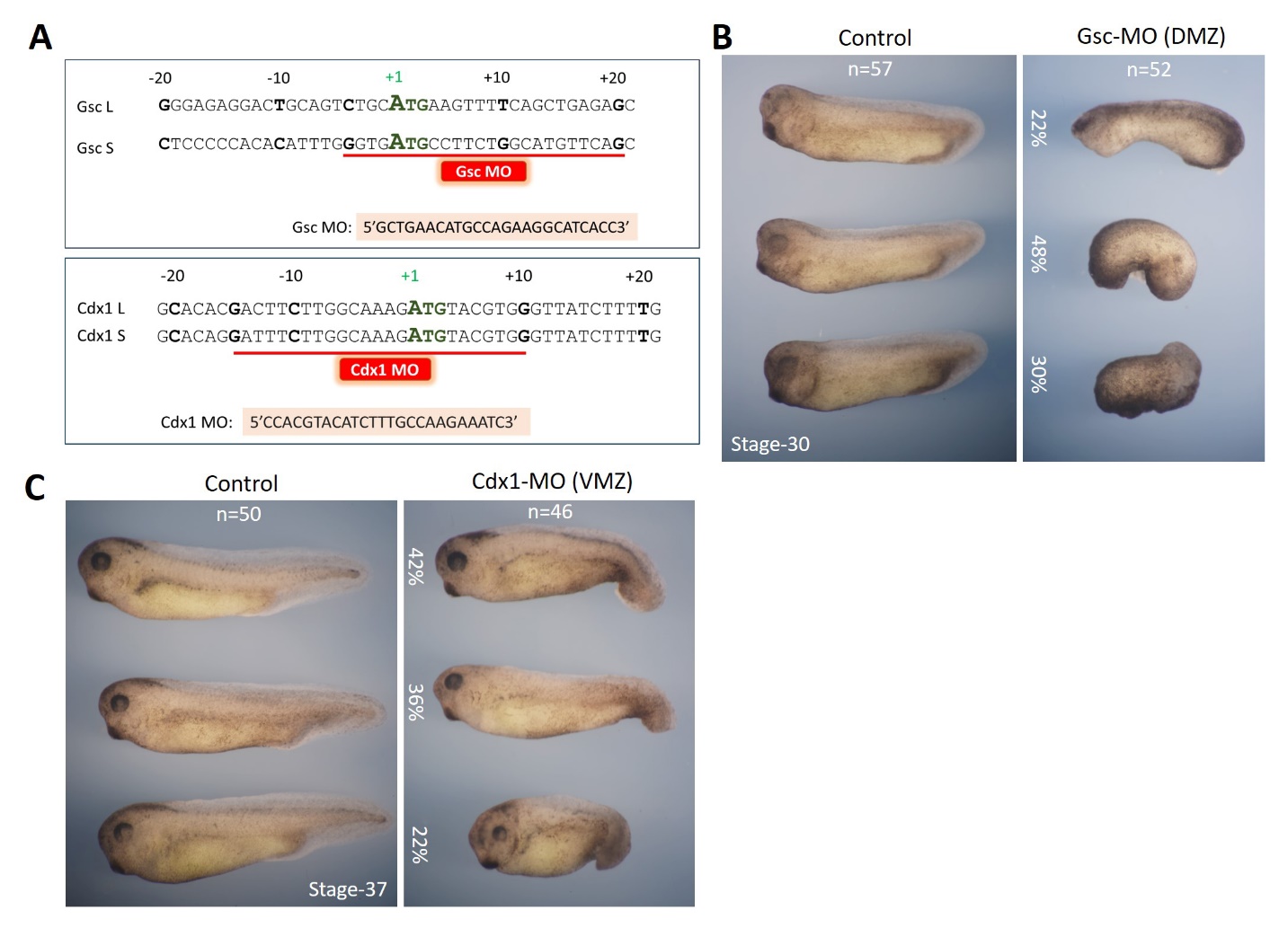


**Supplementary Fig. 3**. Morpholino information and phenotypic effects on *Xenopus.*

1. MO sequences and their target sites in the Gsc and Cdx1 Coding sequence regions..
2. Gsc knockdown phenotype. Gsc MO (70 ng) was injected into each dorsal-

blastomere at the four-cell stage, and its phenotypic effect was observed at NF stage 30.

1. Cdx1 knockdown phenotype. Cdx1 MO (38 ng) was injected into each ventral-

blastomere at the 4-cell stage and the phenotype was observed at NF stage 37.


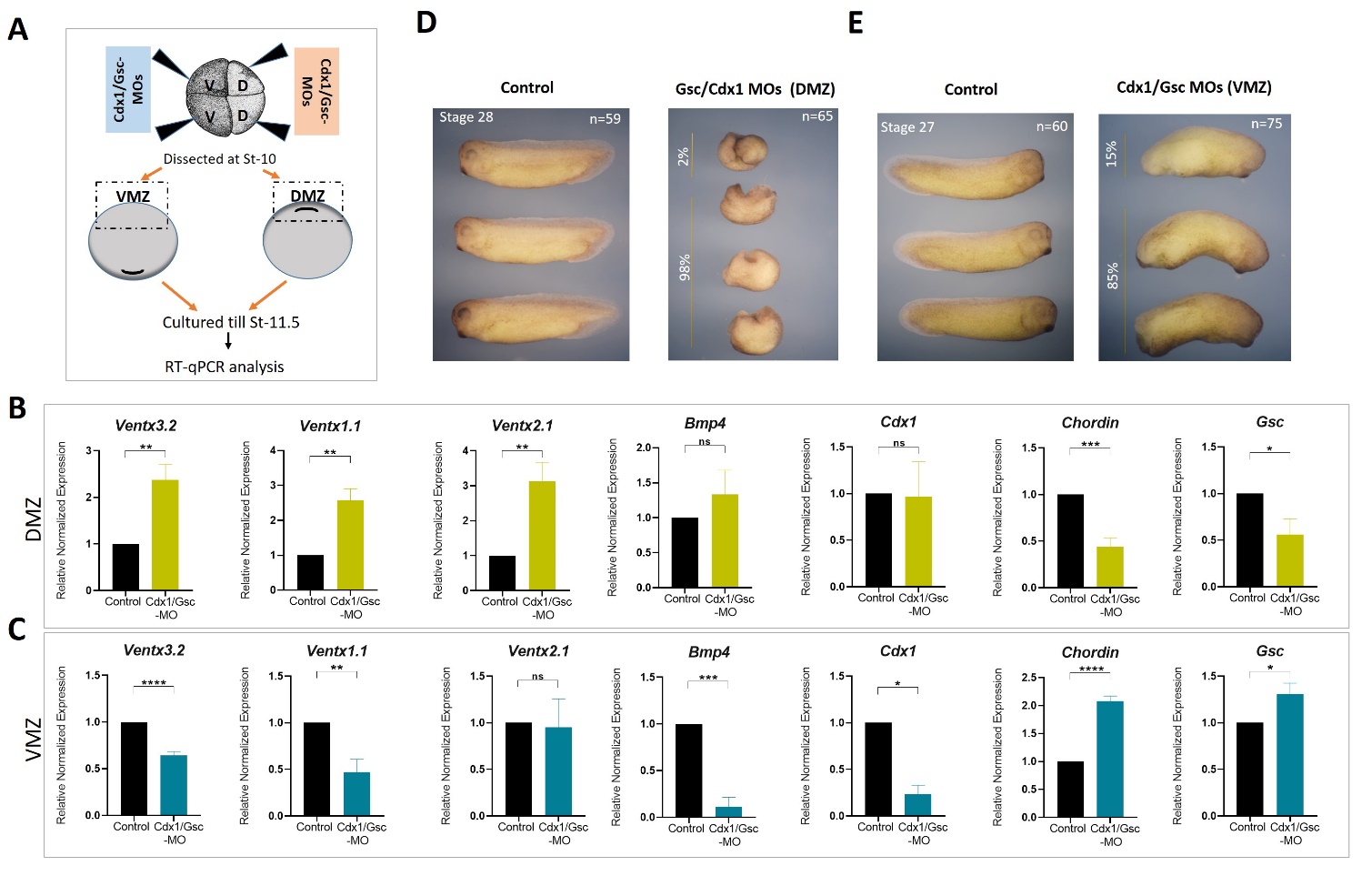


**Supplementary Fig. 4**. Double knockdown of Cdx1 and Gsc in *Xenopus laevis*.

1. Embryos were injected dorsally (D) with 70ng Gsc MO and 20ng Cdx1 MO and ventrally (V) with 38ng Cdx1 MO and 40ng Gsc at 4-cell stage. DMZ and VMZ explants were dissected at stage 10 and were culture till stage 11-11.5.
2. qRT-PCR analysis of Cdx1/Gsc depleted DMZ. blastomere at the four-cell
3. qRT-PCR analysis of Cdx1/Gsc depleted VMZ
4. Phenotype of Cdx1/Gsc-MO dorsally injected.
5. Phenotype of Cdx1/Gsc-MO ventrally injected.

1. [↑](#footnote-ref-1)
