# Supplementary material for: Cdx1 and Gsc distinctly regulate the transcription of BMP4 target gene ventx3.2 by directly binding to the proximal promoter region in Xenopus gastrulae
Source: Mol Cells. 2024 Mar 23;47(4):100058. doi: 10.1016/j.mocell.2024.100058 (PMC11031840; doi:10.1016/j.mocell.2024.100058)
Supplement: Supplementary file 2 — Supplementary material. [file mmc2.docx]

**All members of the Ventx family of *Xenopus laevis* protein sequences (with the exception of Ventx3.1) belong to the L homolog, which is known to be functional. The L homolog protein for Ventx3.1 has not yet been found. Homeodomains highlighted in yellow color.**

>XBmRNA57150 (Ventx1.1L)

MVQQGFSIDLILARSREKAADGKDSMSSRPHIPCAPQPLPPNKYAKEMPRRKDGQDVQEHSSCSLGEQGKKLQYSSLSSAALHRSWGSSDDFSSVGSEDDSTEGSPSPMRNSQETETDHSGESPKSDLQRRLRTAFTPQQISKLEQAFNKQRYLGASERKKLATSLRLSEIQVKTWFQNRRMKLKRQIQDQQHNMVPPPVCYPQTFPYYPGVLPVPLNSGSFYQPPAHPFQAPQNSYIPDPRFIPQPLPHHIRMSVALQQQYPPLGLPPGRYFTGLASKNDG*

>XBmRNA57148 (Ventx1.2L)

MVQQGFSIDLILARSREEAADGKDSMSSRPHIPCAPQPLPPTKYAKEMPRRKDLQEHGDITSFQCSSGEQGINRQFPKPSSAVLHRSSGSSDEFSPPDSEDDSTESSGRSSQENDTEQMEKSPKSDLQRRLRTAFTPQQITKLEQAFNKQRYLGASERKKLATSLQLSEIQVKTWFQNRRMKLKRQIQDQQHNLVPPPVCYPQTFPYYPGVLPVPLNSRSFYQPPAHPFQAPHHSYIPQPLHHHIRMSAHQDQYPPFFGARFM*

>XBmRNA57151 (Ventx2.1L)

MTKAFSSVEWLAQSSRRSHREQPSKVDQRYSPYPRPSLPSWNSDVSPSSWNSQLSPDPDSAQVSPCPVSAQVSPYSSDSEISLYSHEEEASFYGMDFNTSSSPGDNGLLHRDTTSYSRGMEAMSASTPATSPVKGAQPVDSAYSTSTDSGYESETSRSNSTAPEGDASVSLSPNDTSDEEGKMGRRLRTAFTSDQISTLEKTFQKHRYLGASERRKLAAKLQLSEVQIKTWFQNRRMKYKREIQDGRPDSYHPAQSFGVYGYAQQPTPVFQHAVQHPYPGYNPLMETLPGTMPYTMHSPAMDSLTPFNSQPFQMLYLPQQHLGQPLTYQEERPFVRY*

>XBmRNA57149 (Ventx2.2L)

MTKAFSSVEWLAQSSRRSHREQPSKVDQKYSPYPSPSLPSWNSDVSPSSWNSQLSPDPDSAQVSPCPASAQVSPYSSDSEISLYSHEEEASFYGMDLNTSSSPGDNGLLHSEMVSVPDNIPRASSDEDAAKSAYSTSTDSGYESETSCSSSTAPEGDAISLSPNDTSYEEGKMGRRLRTAFTSDQISTLEKTFQKHRYLGASERQKLAAKLQLSEVQIKTWFQNRRMKYKREIQDGRPDSYHPAQFFGVYGYAQQPTPVFQHAVQHPYPGYNPLMETLPGTMPYTMHPPAMDSMTPFNSQPFQMLYLPQEHLGQPLTYQEERPFVRY*

>Xelaev18036900m (Ventx3.1S)

MEKTPYLVEWLSKSSQRKPLNINMDLSGYNLGDPDFNKPGNLRSTTSSANSNKKENCGLRNVLNEHGRISVAVQIKSQTSLQESEIPQKVFKEVQPTDQVDRATNITDSRKESSSLSDEEHVSKGRTKFAPEQLEELERSFRENRYIGSNERRQLSKVLKLSERQIKTWFQNRRMKFKRQSQDTRVEAFFSSLYTPSYNYPELSAPAYSVQPELPALVPPPVATSVPPFSCIYPALTPGVRQPIMPSTIYICALPCLFILC*

>XBmRNA57147 (Ventx3.2L)

MEKRPYSVEWLSESSQRKPVYSNVDLLGFKSGYPYKEYSISLPSRATGPTLPCLDYREKENYCARNIQNEERSPPVKDPIQSQPAAQELDTSRRALMMVPASDQSTGDQADKVTGMTDTNKKNKSVCDEDAAARARTKFSAEQLEELERSFKENRYIGSSEKRRLSKVLKLSETQIKTWFQNRRMKFKRQTQDARVEAFFSGLYVPYYGYPDLPTPGYSVQPEFSVLAPHTMAASSVPFSPLQSTVISPGLHPTTIPSANFGSYPCSSMLVHPMLNEPTRQRYSPY*
