# Supplementary figures and images for: Cdx1 and Gsc distinctly regulate the transcription of BMP4 target gene ventx3.2 by directly binding to the proximal promoter region in Xenopus gastrulae
Source: Mol Cells. 2024 Mar 23;47(4):100058. doi: 10.1016/j.mocell.2024.100058 (PMC11031840; doi:10.1016/j.mocell.2024.100058)

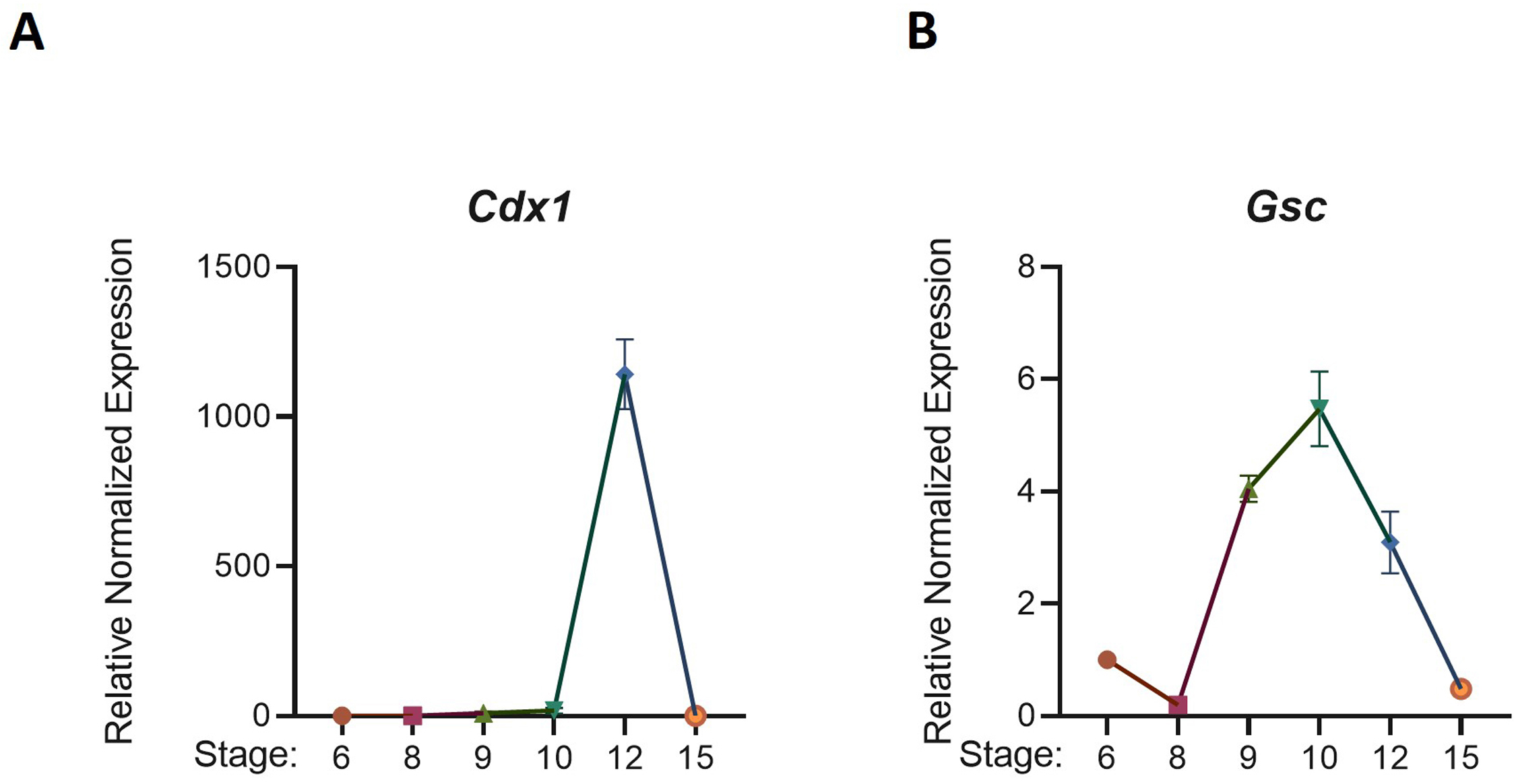

Supplement: Supplementary file 3 — Supplementary material. [file mmc3.jpg]

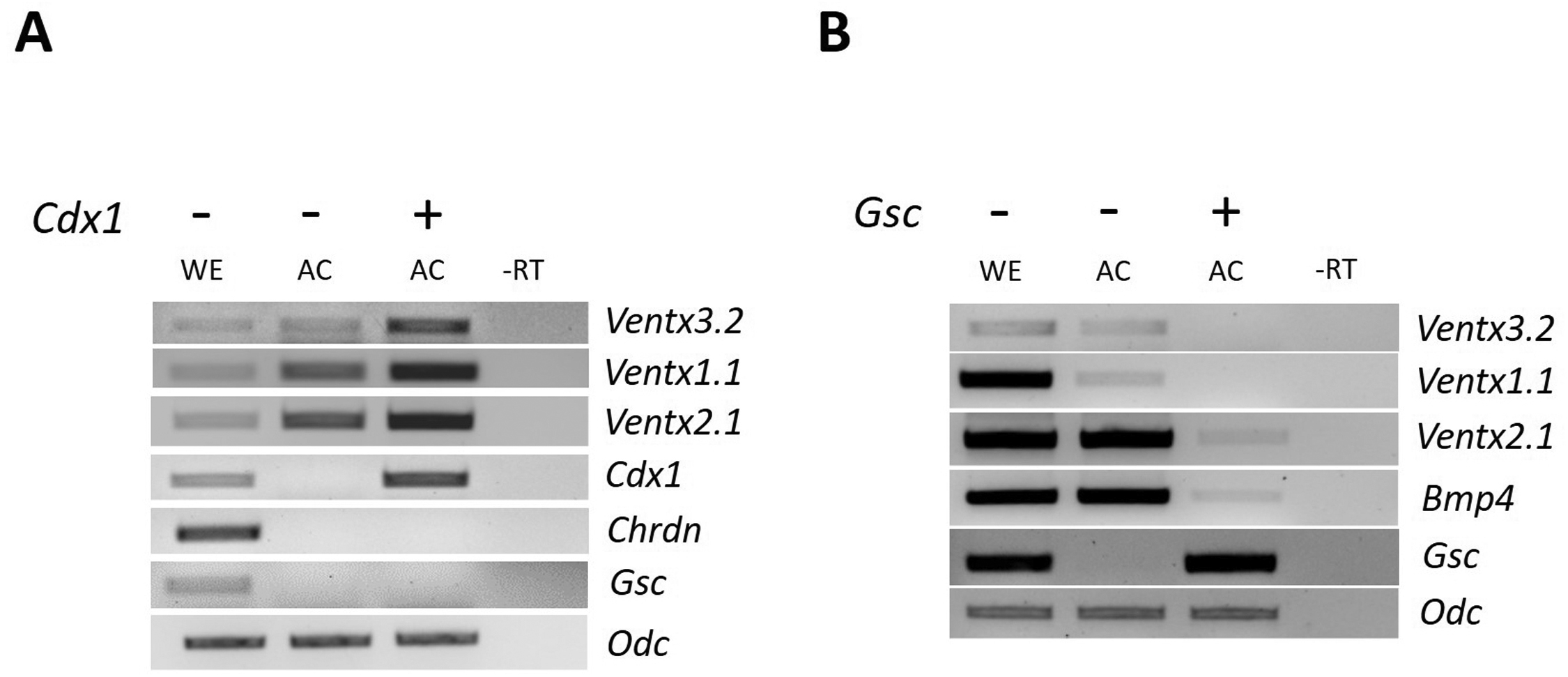

Supplement: Supplementary file 4 — Supplementary material. [file mmc4.jpg]

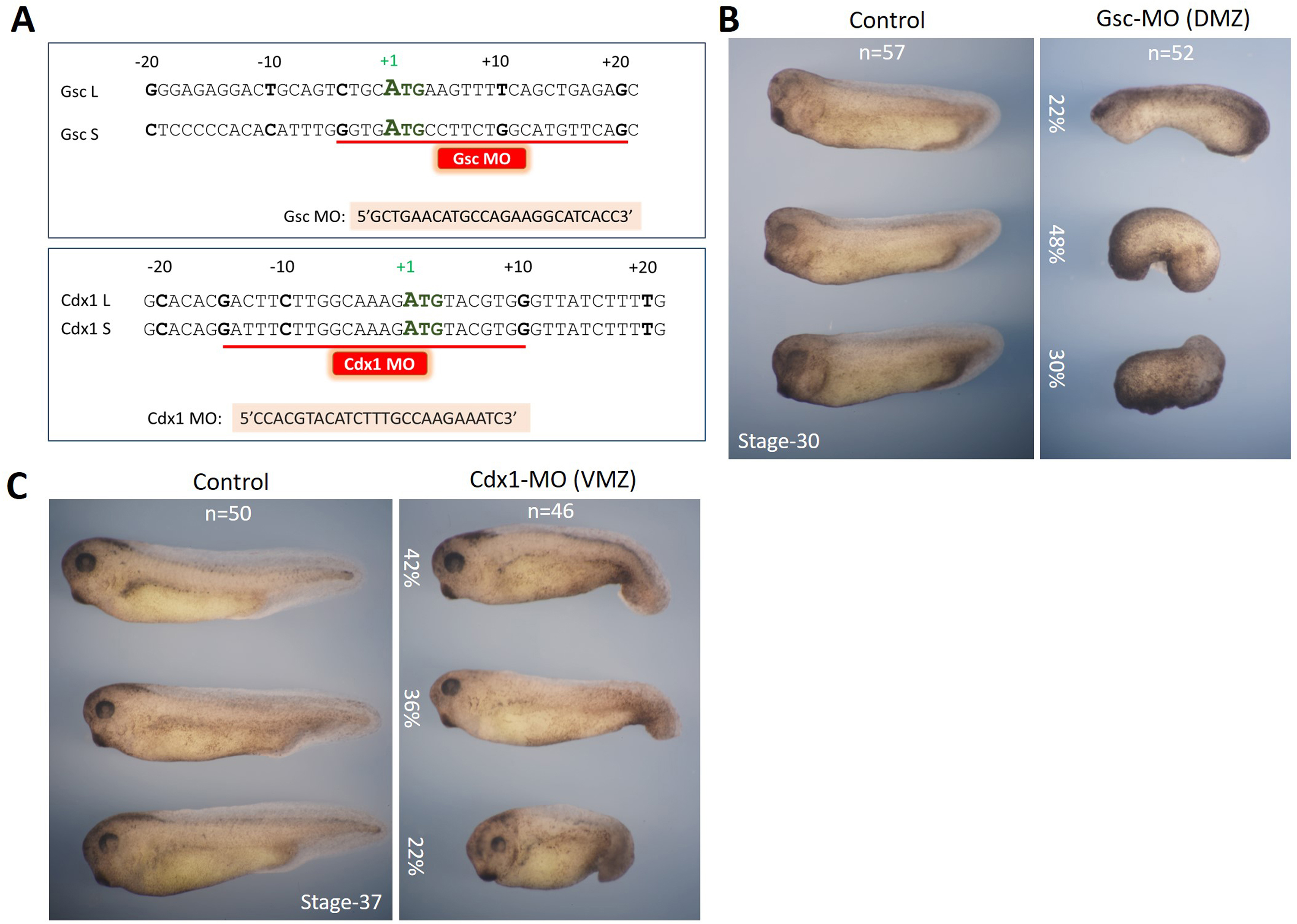

Supplement: Supplementary file 5 — Supplementary material. [file mmc5.jpg]

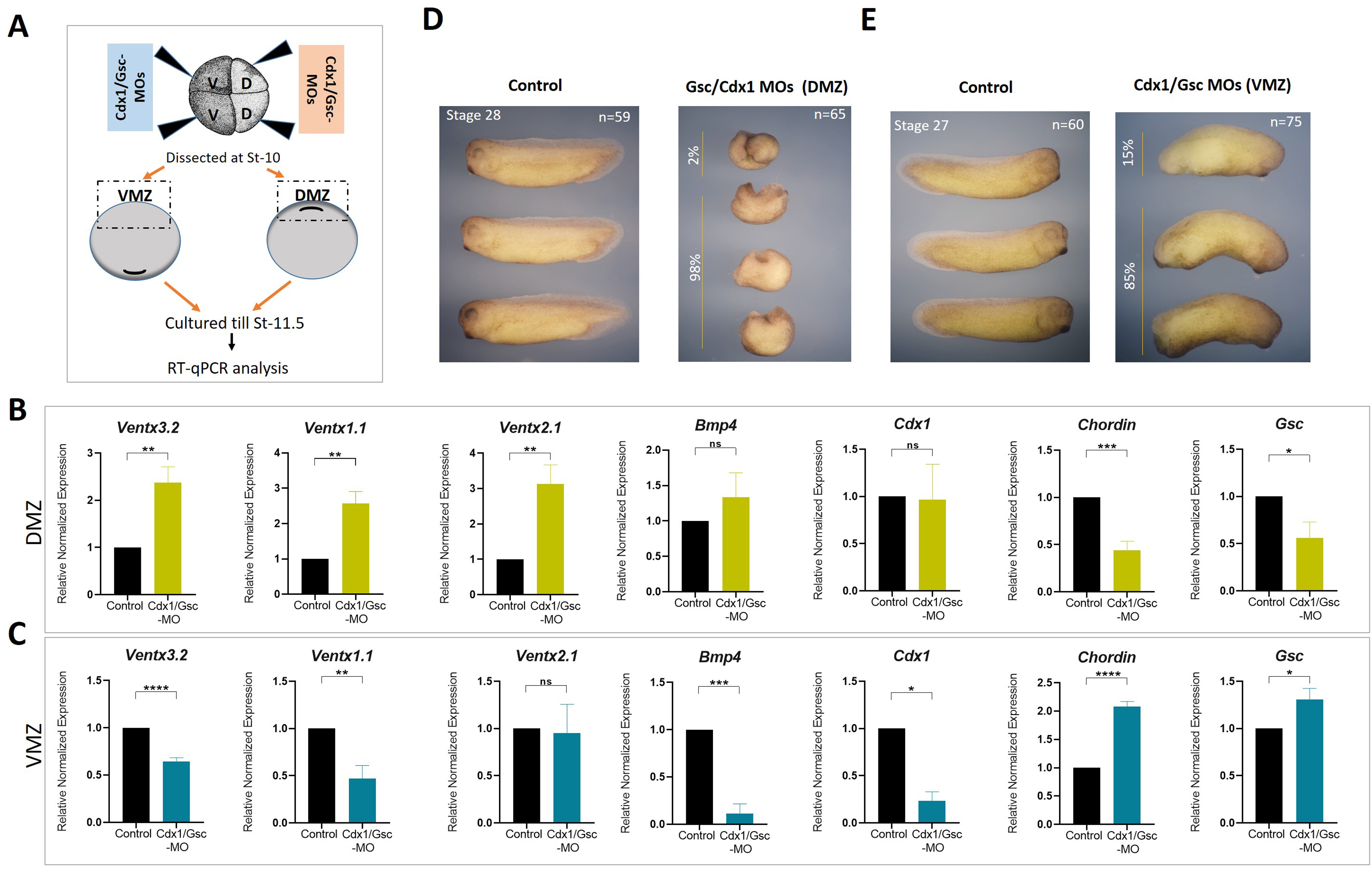

Supplement: Supplementary file 6 — Supplementary material. [file mmc6.jpg]
